# Establishment and application of a rapid visual diagnostic method for *Streptococcus agalactiae* based on recombinase polymerase amplification and lateral flow strips

**DOI:** 10.1038/s41598-024-56138-7

**Published:** 2024-05-02

**Authors:** Tuo Ji, Ye Cai, Yuzhi Gao, Gang Wang, Yongchang Miao, Xuzhu Gao

**Affiliations:** 1 Institute of Clinical Oncology, The Second People’s Hospital of Lianyungang City (Cancer Hospital of Lianyungang), Lianyungang, China; 2 Institute of Clinical Oncology, The Second People’s Hospital of Lianyungang Affiliated to Bengbu Medical University, Lianyungang, China

**Keywords:** Recombinase polymerase amplification, Lateral flow strips, *Streptococcus agalactiae*, Biological techniques, Microbiology

## Abstract

This study aims to establish a rapid diagnostic method for *Streptococcus agalactiae* (GBS) based on recombinase polymerase amplification (RPA) and lateral flow strips (LFS). The best primer pairs designed by SIP gene were screened according to the basic RPA reaction, then the probe was designed. The reaction condition was optimized based on the color development of the LFS detection line. To ascertain the reaction specificity, 10 common clinical pathogens and 10 clinical specimens of GBS were tested. Furthermore, the reaction sensitivity was assessed by utilizing a tenfold gradient dilution of GBS genomic DNA as templates. RPA–LFS method was compared to the qPCR assay and biochemical culture method for the Kappa consistency test. The RPA–LFS technique was able to complete the amplification process within 30 min and the results were observed on lateral flow strips. The method is highly sensitive, with a minimum detection limit of 1.31 ng for GBS. The RPA–LFS method showed consistent accuracy of results compared to qPCR and the culture–biochemical method. The establishment of this method is conducive to the development of on-site immediate detection, which can provide information for the timely development of a reasonable antimicrobial treatment plan, and has a greater potential for clinical application.

## Introduction

*Streptococcus agalactiae* (GBS) is a beta-hemolytic gram-positive streptococcus^1^. GBS strains are the only streptococcal species containing the Lancefield group B cell wall-specific polysaccharide antigen^2^. GBS commonly colonizes the rectovagina and causes infectious diseases in newborns. It is also becoming a cause of disease in non-pregnant adults^3^. In the elderly, immunocompromised individuals, and infants, GBS causes serious infections such as septicemia, pneumonia, and meningitis^4^. Neonatal GBS infections can be categorized as early-onset and late-onset infections. Symptoms of early-onset GBS appear in the first week of life and thereafter late-onset. Perinatal transmission of GBS to the neonate through the birth canal is a major factor in early-onset neonatal infections^5^, whereas delayed-onset GBS is an important cause of sepsis and meningitis in infants^6^. Currently, the colonization rate of *S. agalactiae* in pregnant women is 11.3% in China^7^, which is not high compared with foreign countries, but the severity of its infection causing illness in pregnant women and newborns makes early screening and timely treatment of this bacterium particularly important. Routine GBS screening is accomplished by polymerase chain reaction (PCR)-based assays and culture. Bacterial culture is currently recognized at home and abroad as an important method for detecting GBS and is the gold standard for detecting GBS. It has good sensitivity and specificity, but the method is time-consuming and complicated to operate^8^, which is not favorable for the immediate detection of pathogenic bacteria. Although real-time PCR assays and other molecular biology techniques have been developed and commercialized, their use in most laboratories is limited due to the required investment and expertise^2^. Therefore, the establishment of a convenient, rapid, and sensitive assay is essential for the rapid diagnosis of *S. agalactiae*.

**Figure 1:**
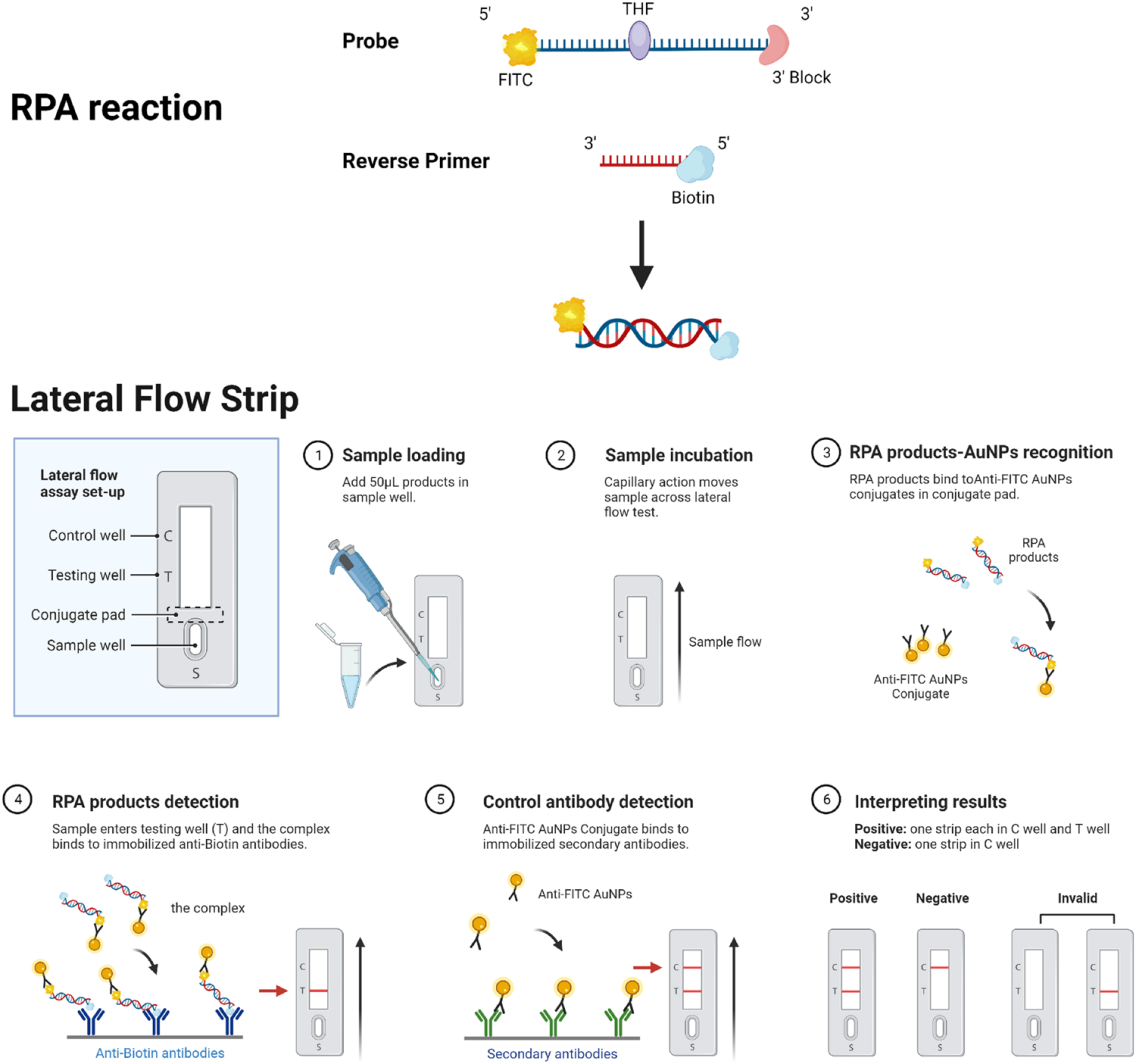
Schematic diagram of the RPA–LFS assay.

Recombinase polymerase amplification (RPA) is an instrument-independent technique for rapid amplification of nucleic acids at 37–42 °C. The technique is effective for a wide range of applications. Common isothermal amplification techniques include Helicase-dependent amplification, Nucleic acid sequence-based amplification, recombinase polymerase amplification, and Loop-Mediated Isothermal Amplification, etc. Compared with PCR and other isothermal amplification methods, RPA has the advantages of simple operation, fast reaction speed, and low equipment requirements^9^. RPA consists of three key enzymes: DNA polymerase (DNA pol), T4 phage uvsX recombinase and cofactor uvsY, and single strand DNA-binding protein (SSB). Firstly, the RPA reaction was carried out under ATP-fed conditions, and the recombinase protein uvsX bound to the RPA primer with the help of cofactor uvsY to form a recombinase-primer complex. Subsequently, the complex binds to homologous sequences in the reaction system, forming a D-loop structure from which the recombinase uvsX detaches. Finally, the target gene fragment is amplified in the presence of DNA polymerase. The replaced DNA strand binds to SSB to prevent further replacement. Repeat the above steps to achieve exponential amplification of the target gene. Generally, RPA amplification products can be detected by agarose gel electrophoresis, probe-based fluorescence real-time system, and lateral flow strips (LFS)^10^. LFS is a paper-based POCT device that uses colloidal gold nanoparticles (AuNPs) as signal labels and is easy to operate^11^. The combination of RPA and LFS technology can break the laboratory constraints for rapid amplification and visualization of target genes in a short time^12^. The principle of the RPA–LFS assay is described in Fig. 1. The RPA–LFS reaction relies on a specific 46–52 oligonucleotide chain probe, modified at the 5′-end with a fluorescent group (FITC or FAM) and 3′-end with a polymerase extension blocking group. 3′-end block group is used to block amplification downstream of the probe. Meanwhile, a tetrahydrofuran residue (THF) is used to replace a nucleotide, then THF will be recognized and cleaved by a nfo nuclease under the RPA–LFS reaction. The reverse primer should be labeled at 5′ end with biotin. The forward primer is a conventional RPA primer. After an RPA reaction, the DNA amplicons will include fluorescent and biotin labels. When the amplified product is added to the LFS, it will bind with the anti-fluorescent antibody labeled by AuNPs on the strip to form a ternary complex. The ternary complex will be captured by anti-biotin antibodies once the contact of the complex and the test line. Plenty of gold nanoparticles will be visible to the naked eye when they accumulate on the detection line.

The RPA–LFS reaction requires the specific probe and the reverse primer. The probe has a THF site inserted in the middle, a blocking group attached at the 3′ end, and a FITC group labeled at the 5′ end. The reverse primer is labeled with biotin at the 5′ end. The RPA–LFS reaction forms a binary complex product with FITC and biotin. When the product is added dropwise to a nucleic acid test strip, the amplification product binds to the AuNPs-labeled anti-FITC antibody, forming a ternary complex. When the ternary complex enters the test line, it gets trapped by the anti-biotin antibody, causing the test line to turn red. Meanwhile, as the amplification product flows toward the control line, the gold standard antibody binds with the secondary antibody, resulting in the coloration of the control line. When both the detection line and the control line are colored, the test result is positive. If only the quality control line is colored, then the result is negative. However, if neither the control line nor the test line are colored, or if the test line is colored but the control line is not, then the data is not available.

Currently, RPA–LFS has been applied to *Pseudomonas aeruginosa*^13^, *Vibrio parahaemolyticus*^14^, and *Candida albicans*^15^ for the detection of these organisms. However, for *S. agalactiae*, RPA–LFS has not been established. This study aimed to establish an RPA–LFS technique for the rapid detection of *S. agalactiae*.

## Results

### Primer and probe design

**Figure 2:**
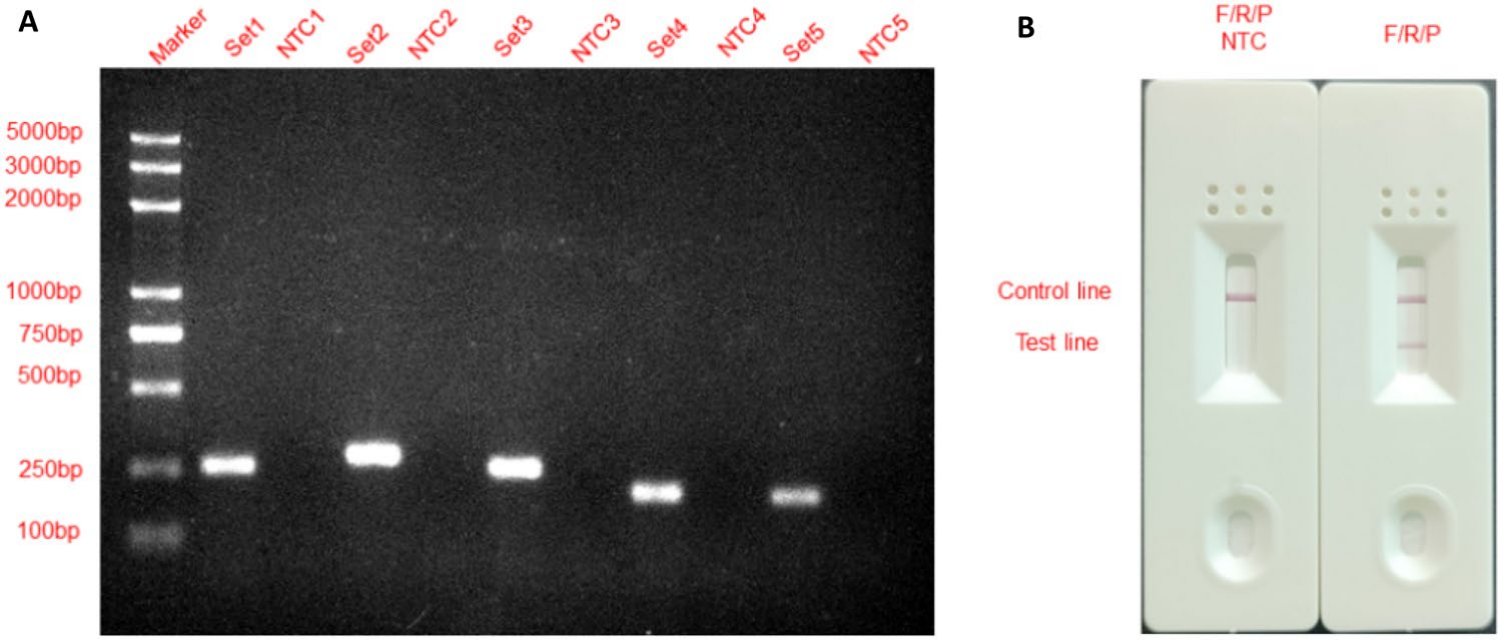
Screening of optimal primer pairs and probe design. (**A**) Agarose gel electrophoresis results of basic RPA products; (**B**) RPA–LFS results after base mismatch.

Five sets of primer pairs were designed (Table 1) and obtained according to the specific sequence of the *S. agalactiae SIP* gene (NC_007432.1:47331-48635). Using *S. agalactiae* genomic DNA as a template, the 5 sets of primer pairs were screened for optimal primer pairs by basic RPA reaction and 2% agarose gel electrophoresis. As shown in Fig. 2A, the five sets of primer pairs produced specific bands consistent with the expected results, and no primer dimers were produced. No bands were produced in the No Template Control (NTC) group. In comparison, primer pair 2 was the brightest in color and no primer dimer was visible to the naked eye, so primer pair 2 was chosen for probe design.

**Table 1:** Primer pairs and probe sequences.

Primer pair	Name	Primer sequence (5′-3′)	Primer length (bp)	Product length (bp)
Set1	GBS-F1	CAGTACATACCGTCCGGGAGATCCAGGTGA	30	259
	GBS-R1	TAAGTGAACGTGGTTATAGTGGTTGGCAGT	30	
Set2	GBS-F2	GTTTCTCCAATGACGACATAATCTTCTGCG	30	292
	GBS-R2	CTTGAACACTTGCCACTCTACGGGCTGCTA	30	
Set3	GBS-F3	CGGTAAGAACTGTAGCAGCCCCTGGAGTGG	30	264
	GBS-R3	GAGGTTGGAGCCTTGGATTTTCAGGATGTG	30	
Set4	GBS-F4	ACGATTGTTTCGCCACTGACGACACATTCT	30	210
	GBS-R4	TGGTGATACTGTTGTTGACTGACTGACTGA	30	
Set5	GBS-F5	GTTTCGCCAATGATGACATATTCTTCTGCG	30	204
	GBS-R5	TGGTGATACTGTTGTTGACTGACTGACTGA	30	
P		**FITC-**GTTTCTCCAATGACGACATAATCTTCTGCG**[THF]**CAGCTTTGAAATCAA-**/C3-spacer/**	45	–

F, R, and P represent forward primer, reverse primer, and probe, respectively. The underline represents the modified base. The bold font represents modified groups.

Probes were designed based on the upstream primers of primer pair 2, and the amplification performance of the primer–probe combination F/R/P and the presence of false positives were examined by RPA–LFS. Considering that unfavorable primer interactions may occur in the RPA reaction environment, low molecular weight DNA by-products (< 100 bp), i.e. “primer noise”, were generated. To reduce the generation of “primer noise”, we introduced base mismatches into the primers. The mismatch principles were as follows: base mismatch was performed in regions with more than four consecutively paired bases; three bases near the 3′ end could not be replaced; no more than five bases could be replaced in each primer; no consecutive two-base substitutions were allowed; and priority was given to the use of A-G swaps and T-C swaps. The results are shown in Fig. 2B. After the introduction of appropriate base mismatches, the F/R/P primer–probe combination produced red bands on both the LFS detection line and the quality control line, which indicated that the F/R/P primer–probe combination had good amplification performance. In the no-template control group, no red band was shown on the LFS detection line, indicating that there was no false-positive amplification of the F/R/P primer–probe combination after the introduction of the mismatch, and the results were in line with expectations. Subsequent experiments were performed using the F/R/P primer–probe combination.

### Optimization of reaction conditions

**Figure 3:**
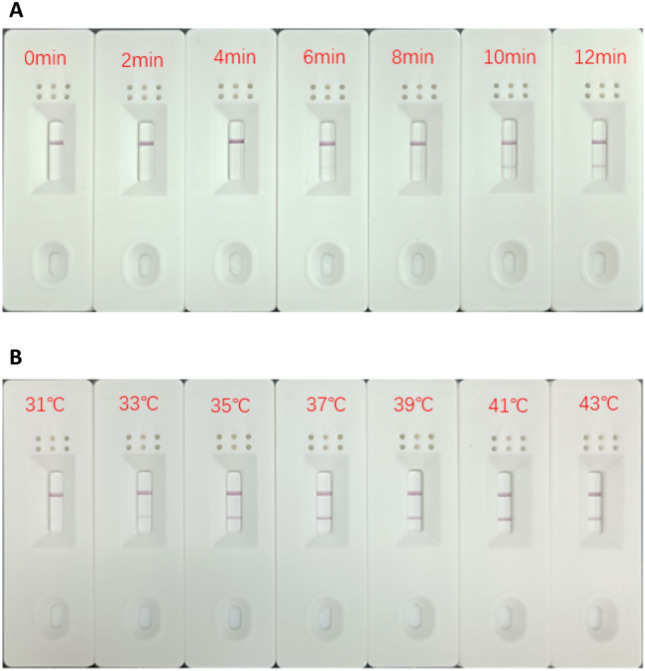
Optimization of reaction time and temperature for RPA–LFS. (**A**) Optimization of the optimal reaction time for RPA–LFS; (**B**) Optimization of the optimal reaction temperature for RPA–LFS.

To optimize the RPA reaction, we adjusted the reaction time and temperature. Firstly, the reaction temperature was controlled at 37 °C and the reaction time was set to 0, 2, 4, 6, 8, 10, and 12 min to observe the results. The results are shown in Fig. 3A, at 6 min the detection line appeared lighter bands; at 10 min the detection line was clear and bright. After 10 min, with the extension of the reaction time, there was no obvious change in the detection line. Next, the reaction time was controlled at 10 min, and the RPA reaction was carried out at a temperature gradient of 31, 33, 35, 37, 39, 41, and 43 °C, respectively, to determine the optimal reaction temperature. As shown in Fig. 3B, a lighter band appeared at a temperature of 31 °C; the band was clear and bright in the range of 35–43 °C. The optimal conditions for the RPA–LFS reaction were a temperature of 35 °C and a reaction time of 10 min, and the subsequent experiments were carried out under these conditions.

### Specificity of the RPA–LFS assay

**Figure 4:**
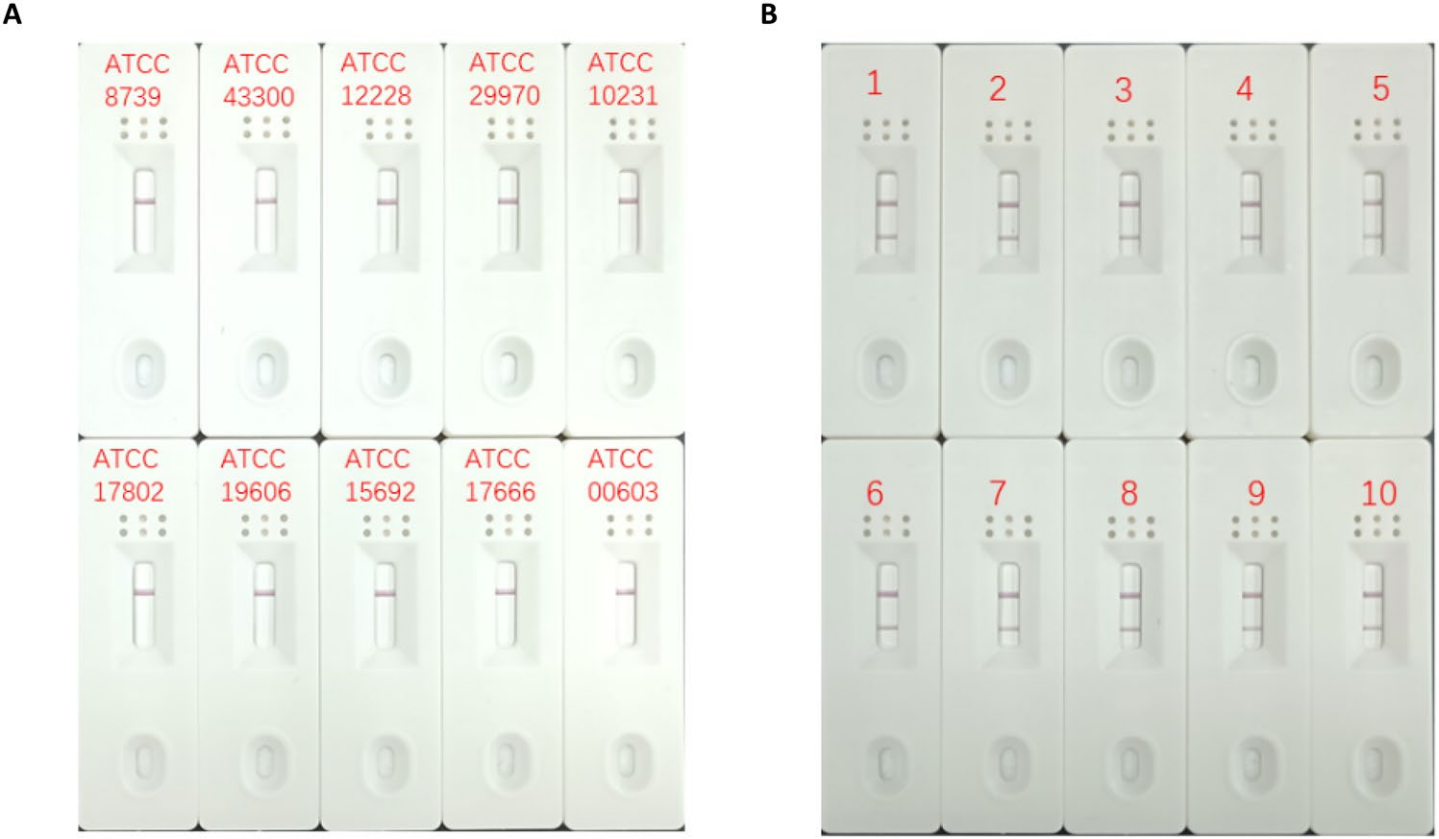
RPA–LFS specific assay. (**A**) The RPA–LFS assay was conducted using 10 standard pathogenic microbial strains; (**B**) The RPA–LFS assay was conducted using 10 clinical specimens of *Streptococcus agalactiae.*

Ten common clinical pathogens and 10 clinical sources of *S. agalactiae* were detected. When the genomes of other pathogenic bacteria were used as templates for the RPA–LFS reaction, no positive signals appeared on the detection line (Fig. 4A); when clinically isolated *S. agalactiae genomic* DNA was used as a template, a clear positive signal appeared on the detection line (Fig. 4B). The results showed that our established RPA–LFS detection system has good specificity for *S. agalactiae* and no cross-reactivity with other pathogenic bacteria.

### Sensitivity of the RPA–LFS assay

**Figure 5:**
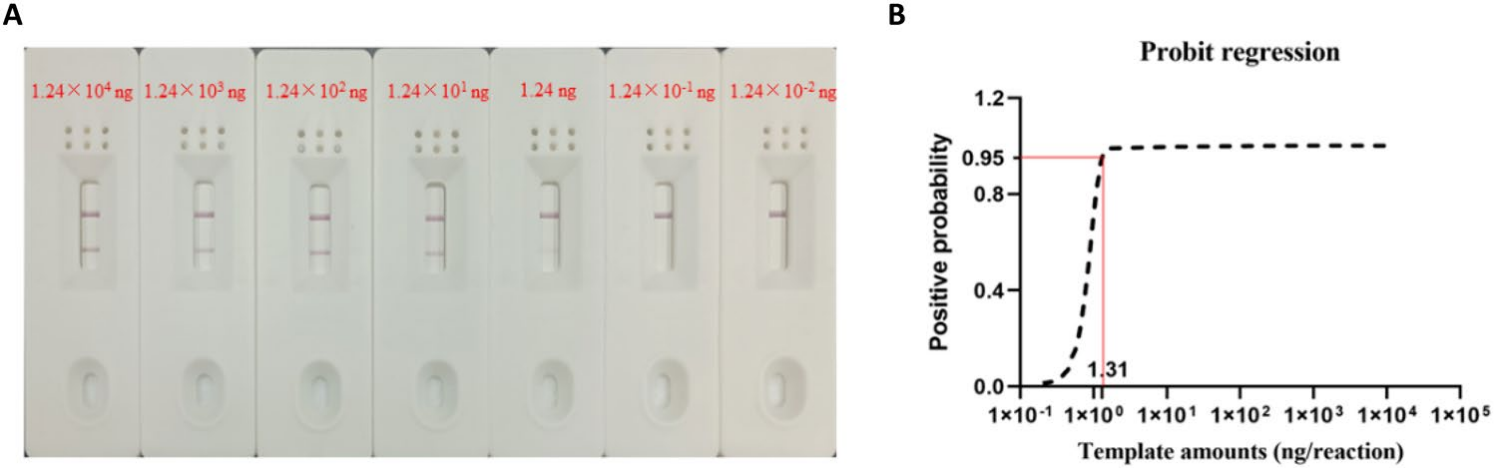
Sensitivity of the RPA–LFS assay. (**A**) Sensitivity of the RPA–LFS system using different concentrations of *Streptococcus agalactiae* genomes; (**B**) SPSS software analyzed the lowest detection limit of the RPA–LFS system, and the red solid line indicates the 95% detection limit.

To assess the minimum detection value of the RPA–LFS method, a gradient dilution of *S. agalactiae* genomic DNA (1.24 × 10^4^, 1.24 × 10^3^, 1.24 × 10^2^, 1.24 × 10^1^, 1.24, 1.24 × 10^–1^, 1.24 × 10^−2^ ng/μL) was used as the template, and 1 μL DNA template was added to the 50 μL reaction system. When the reaction system contained 1.24 × 10^4^ ng genomes, a strong positive signal appeared on the detection line, and the color of the positive bands weakened as the template concentration decreased (Fig. 5A). In addition, to further determine the accurate the Limit of Detection (LOD) of the RPA–LFS assay, 10 independent assays were performed for all concentrations. The results showed 9 positive results when the genome content in the reaction system was 1.24 ng and no positive result when the genome content was 1.24 × 10^−1^ ng. The results of 10 assays were analyzed by probit regression through SPSS software (Fig. 5B). The lowest detection limit of the RPA–LFS reaction was 1.31 ng at a 95% probability of positivity.

### Application of RPA–LFS in clinical specimen testing

The practical application value of the RPA–LFS method in clinical practice was assessed by comparing it with qPCR and traditional microbiological culture methods (Table 2). The results of 105 clinical specimens collected showed that 22 of 105 specimens tested by the RPA–LFS method with the qPCR method showed positive *S. agalactiae*, whereas only 20 cases were detected as positive by the microbiological culture method. Our established RPA–LFS method showed 100% compliance with the qPCR method and 98.10% positive compliance with the traditional culture–biochemical method, with a Kappa index of 0.9405.

**Table 2:** Assay performance of different methods.

Methodologies	Positive/case	Negative/case	Total number/case	detection time
RPA–LFS	22	83	105	< 20 min
qPCR	22	83	105	90 min
Biochemical–culture method	20	85	105	2 days

## Discussion

Immediate and precise diagnosis of pathogenic microorganisms is one of the difficulties that cannot be ignored in clinical testing. PCR is one of the most specific and sensitive methods reported for the detection of pathogenic microorganisms. In recent years, the continuous development of molecular biology technology, especially the emergence of PCR technology, has vastly promoted the progress of pathogenic bacteria detection technology. PCR has become the mainstream life science and technology of nucleic acid quantitative^16^. *Streptococcus agalactiae* is reported to be the most common reproductive tract pathogen and is the leading cause of neonatal pneumonia deaths, perinatal infections in pregnant women and their newborns, and sepsis and meningitis in infants and children^17^. The 2020 American College of Obstetricians and Gynecologists publication Preventing Early-onset Group B Streptococcal Disease in Newborns: an ACOG Committee Consensus states that bacterial culture is the current gold standard for GBS detection and recommends that all pregnant women at 36–37 weeks of gestation, regardless of the mode of delivery to be used, be screened for *S. agalactiae* and that antibiotic prophylaxis be instituted for patients who have a positive screening to reduce the incidence of Risk of neonatal infection with *S. agalactiae*^18^. Routine GBS screening is accomplished by PCR and Biochemical–culture method^19^, but neither of them is suitable for POCT. Therefore, this study developed a rapid, convenient, and sensitive diagnostic method based on the RPA–LFS technology to achieve POCT for *S. agalactiae*.

This study used the SIP gene-specific conserved sequence of *S. agalactiae* as a template to design RPA primers and probes. In the process of designing the probes, we found that the primer dimer produced by the downstream primers and probes would lead to the occurrence of false positive signals, so to avoid false positive signals, we introduced base mismatches in our experiments. It has been reported in the literature that the RPA reaction allows the introduction of a few mismatched bases^20^, but the introduction of mismatches at the 3′ end should be avoided. After the introduction of mismatched bases, the primer–probe sequences F/R/P suitable for specific testing of GBS were established in this study. By adjusting the time and temperature, the optimal reaction conditions for the RPA–LFS reaction were determined to be a time of 10 min and a temperature of 35 °C. The optimal reaction conditions for the RPA–LFS reaction were determined by adjusting the time and temperature. Using the gradient-diluted GBS genome as a template, the lowest limit of detection of GBS by RPA–LFS was 1.31 ng, and the sensitivity was not interfered by the genomes of other species. By testing 105 microbiological samples originating from the clinic, our established RPA–LFS method was consistent with the qPCR results.

The goal of this study was to develop a rapid, sensitive, and applicable RPA–LFS assay for *S. agalactiae*, which breaks down laboratory limitations and can be used in environments with limited conditions. However, the method still has some limitations: (1) Low detection flux, which makes it difficult to achieve the detection of large-scale samples; (2) although all the detection processes are free from laboratory limitations, the sample processing and genome extraction still require experimental equipment; (3) LPS is prone to aerosol contamination when detecting RPA amplification products. Nonetheless, our established RPA–LFS method still has a broad application prospect for the detection of *S. agalactiae* infections.

## Materials and methods

### Materials

#### Source of strains

The standard strains used in this study are listed in Table 3, all provided by the Second People's Hospital of Lianyungang City. All standard strains were stored in an ultra-low temperature refrigerator at − 80 °C and activated in a microbiological incubator at 35 °C before use. To verify the applicability of the RPA–LFS technique to clinical specimens, 105 clinical test samples were collected between January and December 2022, of which 66 were from vaginal secretions, 28 from urine, and 11 from blood.

**Table 3:** Standard strains used in this study.

strains	ID number
*Streptococcus agalactiae*	ATCC 12386
*Escherichia coli (E. coli)*	ATCC 8739
*Staphylococcus aureus*	ATCC 43300
*Staphylococcus epidermidis*	ATCC 12228
*Staphylococcus haemolyticus*	ATCC 29970
*Candida albicans*	ATCC 10231
*Vibrio parahaemolyticus*	ATCC 17802
*Acinetobacter baumannii*	ATCC 19606
*Pseudomonas aeruginosa*	ATCC 15692
*Stenotrophomonas maltophilia*	ATCC 17666
*Klebsiella pneumoniae*	ATCC 00603

#### Main instruments and reagents

Microbial Incubator (BluePard, China), Shaking Incubator (Shanghai Minquan, China), Ultra-micro Spectrophotometer (Quawell, USA), Electrothermal Thermostatic Bath (Shanghai Jinghong, China), Electrophoresis Instrument (Bio-Rad, USA), Fully Automated Gel Imaging Analyzer (Shanghai Peiqing, China), Bacterial Genome Extraction Kit (Beijing Tiangen, China), Basic RPA kit (AmpMirae, China), nfo RPA kit (AmpMirae, China), Lateral flow test strips (AmpMirae, China).

### Methods

All methods were performed in accordance with the relevant guidelines and regulations.

#### Bacterial culture and genomic DNA extraction

The strains were inoculated on blood agar plates and incubated for 18–24 h. Single colonies on the blood plates were picked and placed in 5 mL of Luria–Bertani medium, and incubated for 12 h in a shaking incubator at 35 °C. Genomic DNA of *S. agalactiae* was extracted using a bacterial DNA extraction kit and stored at − 20 °C. The DNA concentration was determined by spectrophotometry. Other pathogenic bacteria and clinically acquired *S. agalactiae* were used to extract genomic DNA by high-temperature lysis. After the strains were cultured, a small number of colonies were picked in 100 μL of Tris–EDTA and heated and boiled at 100 °C for 30 min to fully release the genome. After the genome extract was cooled down, and centrifuged at 12,000 rpm for 1 min, the supernatant was taken and stored at − 20 °C for spare.

#### Primer design and RPA reaction

The *SIP* gene sequence of *S. agalactiae* was retrieved from the National Centre for Biotechnology Information (NCBI). Primer pairs were designed using Primer premier 5 software and synthesized by General Biologicals Ltd (China). The RPA reaction was performed using the basic RPA kit. The RPA reaction system consisted of 50 μL: 29.5 μL of reaction buffer, 2.4 μL of 10 μM upstream primer (F), 2.4 μL of 10 μM downstream primer (R), 13.2 μL of genomic template, and 2.5 μL of 280 nM magnesium acetate solution (MgOAc). The components were added sequentially to the reaction tube containing the enzyme components and mixed thoroughly by turning them up and down. Immediately after transient centrifugation, the reaction mixture was incubated in a 37 °C water bath for 30 min. After the RPA reaction was completed, the RPA amplification products were extracted using an equal volume of phenol–chloroform-isoamyl alcohol (25:24:1) mixture. Centrifugation was performed at 12,000 rpm for 5 min, and the supernatant was removed. An appropriate amount of DNA loading buffer was added to the reaction tube, and the RPA reaction products were visualized by 2% agarose gel electrophoresis.

#### Probe design and RPA–LFS detection

After screening the best primer pairs according to the RPA reaction and 2% agarose gel electrophoresis, the probe sequence was obtained by extending the upstream primer 16 bp towards the 3′ end. The 5′ end of the probe was labeled with FITC, the 3′ end was modified with a C3-block, and one base in the middle of the probe was substituted with tetrahydrofuran, of which at least 30 bp was located in the 5′ end of the THF site, and 15 bp to the 3′ end. The 5' end of the downstream primer was labeled with biotin. The RPA–LFS reaction was detected using the nfo RPA kit. The RPA–LFS reaction system consisted of 50 μL of reaction solution: 29.4 μL of reaction solution, 2 μL of 10 μM upstream primer, 2 μL of 10 μM downstream primer, 0.6 μL of 10 μM probe, 13.5 μL template mixture (the total volume of the genome template and ddH_2_O was 13.5 μL) and 2.5 μL 280 nM magnesium acetate solution. The components were added sequentially to the reaction tube containing the enzyme components and mixed thoroughly by turning them up and down. After instantaneous centrifugation, the reaction tube was incubated in a 37 °C water bath for 10 min. At the end of the reaction, 10 μL of the reaction product was taken and diluted 20-fold using double-distilled water, and 50 μL of the diluted solution was added dropwise to the sample hole of the test strip and waited for the color to develop.

#### Specificity test

To test the specificity of the RPA–LFS system, we used the method to test 10 other common clinical standard strains and 10 clinical sources of *S. agalactiae*, as described previously.

#### Sensitivity test

A tenfold gradient dilution of *S. agalactiae* genomic DNA (1.24 × 10^4^, 1.24 × 10^3^, 1.24 × 10^2^, 1.24 × 10^1^, 1.24, 1.24 × 10^−1^, 1.24 × 10^−2^ ng/μL) using double-distilled water was used to test the sensitivity of the RPA–LFS system. 1 μL of genomic DNA dilution was added to the RPA–LFS reaction tube, and double-distilled water was made up to 13.5 μL, while other components remained unchanged. Sequential additions were made such that each reaction contained 1.24 × 10^4^, 1.24 × 10^3^, 1.24 × 10^2^, 1.24 × 10^1^, 1.24, 1.24 × 10^−1^, 1.24 × 10^−2^ ng of *S. agalactiae* genome, respectively, and the rest of the steps were performed as described previously. To further determine the LOD of the method, i.e., the lowest concentration at which there is a 95% probability of detecting a positive product, 10 independent experiments were performed for each set of concentrations. Then probabilistic regression analyses were performed on the data obtained from the 10 independent experiments.

#### Clinical specimen testing

The practical application of the RPA–LFS method in clinical specimen testing was evaluated by comparing it with microbiological culture and qPCR assays. Clinical specimens for microbiological culture and qPCR assays were cultured in selected media and incubated in a microbiological incubator at 35 °C for 18–48 h. Strain identification was carried out on a VITEK 2 automated microbiological analysis system. The qPCR primers were designed according to the gene sequences, and the clinical samples were detected by qPCR according to the operation procedure. The forward primer for qPCR is 5′-GCGTTCCGGTAGCACAAAAA-3′, and the reverse primer for qPCR is 5′-TGAGGTTGGAGCCTTGCATT-3′. qPCR steps were as follows: pre-denaturation at 95 °C for 2 min and then enter the cycling stage, denaturation at 95 °C for 10 s, annealing at 56 °C for 50 s, extension at 72 °C for 15 s, a total of 40 cycles. According to the formula:(number of common positive samples + number of common negative samples) ÷ total number of samples × 100%, the compliance rate of the RPA–LFS method with the other two methods was calculated, and the consistency of the method with the other methods was evaluated by Kappa index.

### Ethics approval and consent to participate

The study was approved by the Institutional Review Board (or Ethics Committee) of The Second People’s Hospital of Lianyungang City (2023–044). We confirm that all experimental protocols were approved by Institutional Review Board (or Ethics Committee) of The Second People’s Hospital of Lianyungang City and every human participant provided their informed consent.

### Institutional review board statement

The study was approved by the Ethics Committee of the Second People’s Hospital of Lianyungang (2023-044).

### Supplementary Information

Supplementary Information 1.

Supplementary Information 2.

## Data Availability

The data that support the findings of this study are available from the corresponding author upon reasonable request.
