# Supplementary material for: Establishment and application of a rapid visual diagnostic method for Streptococcus agalactiae based on recombinase polymerase amplification and lateral flow strips
Source: Sci Rep. 2024 May 2;14:10064. doi: 10.1038/s41598-024-56138-7 (PMC11066032; doi:10.1038/s41598-024-56138-7)
Supplement: Supplementary file 2 — Supplementary Information 2. [file 41598_2024_56138_MOESM2_ESM.pdf]

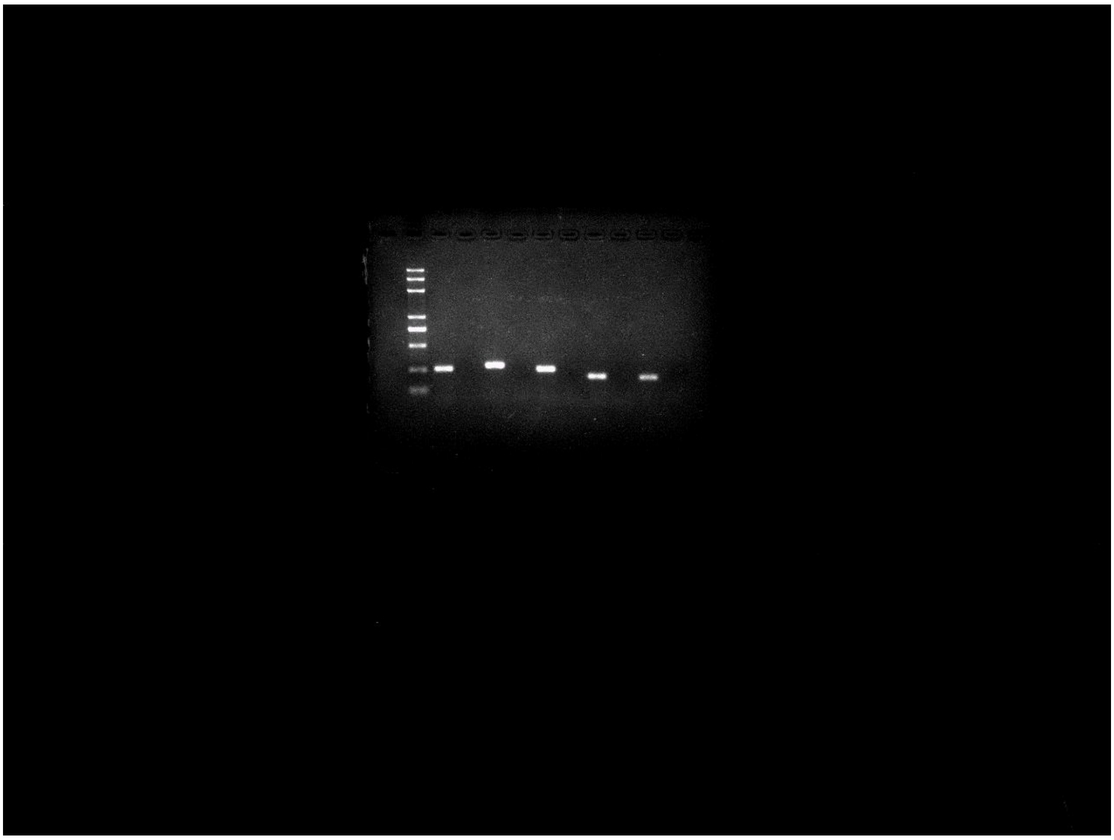

From left to right, it is 1, 2, 3….11 lane

Lane 1: Marker

Lane 2: The RPA result of set 1

Lane 3: The RPA result of no template control 1 (NTC1)

Lane 4: The RPA result of set 2

Lane 5: The RPA result of NTC2

Lane 6: The RPA result of set 3

Lane 7: The RPA result of NTC3

Lane 8: The RPA result of set 4

Lane 9: The RPA result of NTC4

Lane 11: The RPA result of set 5

Lane 10: The RPA result of NTC5
